# Extract from used Xpert MTB/RIF Ultra cartridges is useful for accurate second-line drug-resistant tuberculosis diagnosis with minimal *rpoB*-amplicon cross-contamination risk

**DOI:** 10.1038/s41598-020-59164-3

**Published:** 2020-02-14

**Authors:** Rouxjeane Venter, Stephanie Minnies, Brigitta Derendinger, Happy Tshivhula, Margaretha de Vos, Tania Dolby, Ashley Ruiters, Robin M. Warren, Grant Theron

**Affiliations:** 1 DST/NRF Centre of Excellence for Biomedical Tuberculosis Research, SA MRC Centre for Molecular and Cellular Biology, Division of Molecular Biology and Human Genetics, Faculty of Medicine and Health Sciences, Stellenbosch University, Cape Town, South Africa; 2 National Health Laboratory Services, Cape Town, South Africa

**Keywords:** Tuberculosis, Laboratory techniques and procedures, Translational research, Molecular medicine

## Abstract

Xpert MTB/RIF Ultra (Ultra) detects *Mycobacterium tuberculosis* and rifampicin resistance. Follow-on drug susceptibility testing (DST) requires additional sputum. Extract from the diamond-shaped chamber of the cartridge (dCE) of Ultra’s predecessor, Xpert MTB/RIF (Xpert), is useful for MTBDR*sl*-based DST but this is unexplored with Ultra. Furthermore, whether CE from non-diamond compartments is useful, the performance of FluoroType MTBDR (FT) on  CE, and *rpoB* cross-contamination risk associated with the extraction procedure are unknown. We tested MTBDR*sl*, MTBDR*plus*, and FT on CEs from chambers from cartridges (Ultra, Xpert) tested on bacilli dilution series. MTBDR*sl* on Ultra dCE on TB-positive sputa (n = 40) was also evaluated and, separately, *rpoB* amplicon cross-contamination risk . MTBDR*sl* on Ultra dCE from dilutions ≥10^3^ CFU/ml (C_Tmin_ <25, >“low semi-quantitation”) detected fluoroquinolone (FQ) and second-line injectable (SLID) susceptibility and resistance correctly (some SLIDs-indeterminate). At the same threshold (at which ~85% of Ultra-positives in our setting would be eligible), 35/35 (100%) FQ and 34/35 (97%) SLID results from Ultra dCE were concordant with sputa results. Tests on other chambers were unfeasible. No tubes open during 20 batched extractions had FT-detected *rpoB* cross-contamination. False-positive Ultra *rpoB* results was observed when dCE dilutions ≤10^−3^ were re-tested. MTBDR*sl* on Ultra dCE is concordant with isolate results. *rpoB* amplicon cross-contamination is unlikely. These data mitigate additional specimen collection for second-line DST and cross-contamination concerns.

## Introduction

Drug-resistant tuberculosis (TB) remains a global threat^1^. Of 10 million estimated incidence cases reported in 2017, 588 000 were rifampicin-resistant^2^. Of these ~458 000 were multidrug-resistant (MDR). Despite the improved roll-out of rifampicin-resistance testing, many patients are not diagnosed appropriately or started on effective treatment, resulting in huge TB care cascade gaps^3,4^. For example, in South Africa, 84% of patients with drug-resistant TB have access to rifampicin-susceptibility testing, but only 47% of these are started on likely effective treatment^4^. Similarly, in India, only 41% of the MDR-TB burden was diagnosed in 2013 and, of these, just 32% started on treatment^5^. Innovative approaches are needed to ensure more patients receive comprehensive drug susceptibility testing (DST).

Previous work showed that mycobacterial genomic DNA can be recovered from the rear diamond-shaped chamber of used Xpert MTB/RIF (Xpert) cartridges after the test is complete. This diamond cartridge extract (dCE) is useful for downstream testing with the MTBDR*sl* line probe assay (LPA) (Hain Lifescience, Germany), the only World Health Organization (WHO)-endorsed molecular test for second-line drug resistance, and spoligotyping^6^, a method useful for monitoring the molecular epidemiology of TB outbreaks. This additional testing does not require extra specimen collection nor additional downstream DNA extraction, both of which can exacerbate patient loss within the diagnostic care cascade.

As Xpert is a real-time PCR that generates quantitative information, a cycle threshold value (C_T_ <24) was identified at which downstream dCE testing using MTBDR*sl* was successful and fully concordant with MTBDR*sl* results on matching isolates^7^. However, Xpert dCE was not useful for first-line DST using the WHO-endorsed MTBDR*plus* assay, likely due to interference from large numbers of Xpert *rpoB* amplicons. In addition to the dCE approach, others^8,9^ have shown it is possible to test leftover specimen-sample reagent mix remaining after Xpert, however, remnant volume is not always present and DNA extraction and downstream clean-up might still be needed.

Xpert MTB/RIF Ultra (Ultra) recently superseded Xpert as WHO-endorsed frontline molecular test-of-choice for TB and rifampicin resistance^10^. Compared to Xpert, Ultra has higher sensitivity in paucibacillary samples, however, specificity is overall lower^11–13^. Ultra is a different assay compared to Xpert and it is not necessarily given that the extract approach would be feasible on Ultra dCE. We aimed to confirm that Ultra dCE would be useful for second-line DST. Furthermore, we asked if extract from other chambers within the cartridge other than the diamond (i.e., chambers that are likely *rpoB* amplicon-free), may contain DNA. We quantified this DNA using a *Mycobacterium tuberculosis* complex 16S rRNA real time qPCR and evaluated whether this DNA was useful for first-line DST using the FluoroType MTBDR (FT) (Hain Lifescience, Germany) assay^14,15^. A test such as FT could, for example, be used to check for isoniazid mono-resistance or confirm Ultra rifampicin-resistance results.

Lastly, as the cartridge extraction (CE) procedure involves aspirating fluid rich in *rpoB* amplicons, it may represent a source of cross-contamination. We sought to evaluate this risk, both under a prolonged exposure scenario (where collection tubes were purposely exposed during extended batch extractions) and an absolute worst-case scenario (directly adding dCE to a sample later tested by Ultra). Showing that the extracted cartridge approach in Ultra is compatible with MTBDR*sl* and represents minimal *rpoB* amplicon cross-contamination risk would increase the likelihood of implementation, especially as Xpert is in the process of being phased out in lieu of Ultra. In turn, this could reduce both sputum collection requirements for complete DST and time-to-effective-treatment initiation.

## Methods

### Ethics statement

Methods and protocols were carried out in accordance with relevant guidelines and regulations. The study was approved by the Health Research Ethics Committee of Stellenbosch University (N09/11/296) and the City of Cape Town (10570). Permission was granted to use anonymised residual specimens collected during routine diagnostic practice and thus patient informed consent was waived.

### Ultra and Xpert on dilution series of *Mycobacterium tuberculosis* strains

**Figure 1:**
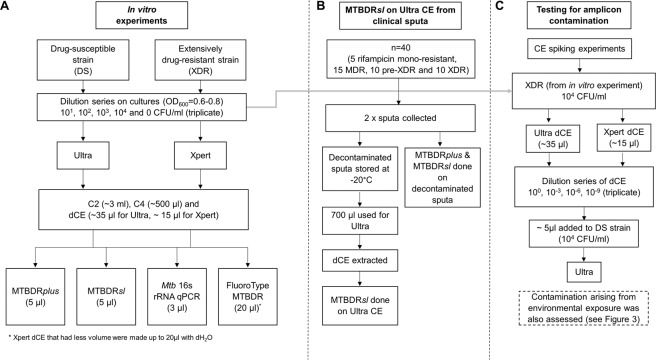
Study flow diagrams for the (**A**) *in vitro* experiment, (**B**) MTBDR*sl* on Ultra CE from clinical sputa experiment, and the (**C**) evaluation of *rpoB* amplicon cross-contamination risk experiment.

Culturing of genotypically-confirmed drug-susceptible (DS-TB) and extensively-drug resistant (XDR) *M. tuberculosis* isolates were done in a Biosafety Level (BSL) 3 laboratory to an OD_600_ of 0.6–0.8 (Fig. 1A). A triplicate tenfold dilution series from three separate cultures [10^0^–10^4^ colony forming units (CFU)/ml] was prepared in phosphate buffer (33 mM Na_2_HPO_4_, 33 mM KH_2_PO_4_; pH 6.8) with 0.025% Tween80 (Sigma-Aldrich, United States). Colony counts were done on 7H11 Middlebrook agar (BD Biosciences, United States). A total of 52 dilutions [four dilutions, 10^1^–10^4^ CFU/ml in triplicate for both strains plus a negative control for each strain; (4 × 3 × 2 + 2) × 2] were made up to 1 ml and tested by Ultra (n = 26) or Xpert (n = 26) per the manufacturer’s instructions^16,17^. Used positive cartridges were stored prior to extraction at 4 °C for ≤3 days. Crude DNA (heat inactivated for 2 hours at 100 °C) from the same strains served as positive controls for downstream tests (16S rRNA gene qPCR, MTBDR*plus*, MTBDR*sl*, FT).

### Ultra on sputum from TB patients

Forty used positive Ultra cartridges done on NALC-NaOH decontaminated sputa from pre-treatment TB patients with known drug resistance [5 rifampicin-mono-resistant, 15 MDR, 10 pre-XDR (resistance to rifampicin, isoniazid and either a fluoroquinolones or a second-line injectable), 10 XDR] were collected from November 2015 to September 2017 and dCEs were extracted as described previously^6^ (Fig. 1B). To confirm MTBDR*sl* results from dCEs, MTBDR*sl* was done per the manufacturer’s instructions directly on corresponding decontaminated sputa^18,19^. Ultra cartridges were processed in a manner blinded to MTBDR*sl* results.

### Recovery of mycobacterial genomic DNA from used Ultra and Xpert cartridges

#### Preparation of work space

BSL2 hood surfaces were sterilised [1% NaOCl (bleach), 70% EtOH, 5 min UV irradiation] before and after each batched extraction. Each cartridge was wiped with 1% bleach and 70% EtOH before and after each extraction.

#### Description of cartridge design

To investigate the feasibility of testing extract from Ultra and Xpert cartridge chambers, an understanding of their design and inner processes is required. As described previously, each cartridge has a similar design consisting of a foot, valve, body, reaction tube and lid^20,21^. The five internal chambers hold buffers and lyophilised PCR reagents used for sputum homogenisation, washing away debris, and DNA extraction, purification, and amplification^22^. The Xpert and Ultra procedures, including the processes inside the cartridges and the contents of each chamber are described in the supplement. After assay conclusion, the volumes typically remaining in each chamber are ~500 µl for Chamber 1 (C1), ~3 ml for Chamber 2 (C2), ~5 ml for Chamber 3 (C3) and ~500 µl for Chamber 4 (C4) [Chamber 5 (C5) had no volume remaining after test completion].

#### Diamond chamber extract

**Figure 2:**
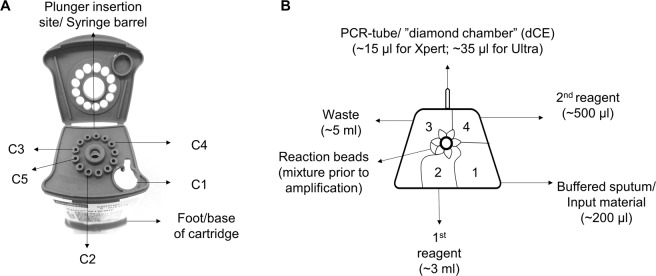
(**A**) Entry points through the lid of the cartridge for access to different cartridge chambers. (**B**) Top-down cross-section of the inside of the cartridge corresponding to the access points.

dCEs were extracted from all positive cartridges by puncturing the rear chamber with a sterile 29 G × 1/2′′ 1 ml insulin syringe (Avacare, South Africa) (Fig. 2A,B) as described previously^6^. The full volume was extracted (~15 µl for Xpert; ~35 µl for Ultra). CEs were stored in microcentrifuge tubes at −20 °C prior to analysis.

#### Other chambers

Five cartridge chambers (C1, C2, C3, C4, C5) were accessed by inserting a 22 G spinal needle (Becton Dickinson, United States) fixed a 5 ml syringe (Fig. 2A; a pipette may also be used for C1) and the entire volume withdrawn (Fig. 2A,B). C5 had no remaining volume left after Xpert or Ultra test completion. No DNA extraction or purification steps were done for downstream assays.

### 16s rRNA gene quantitative PCR (qPCR) on cartridge extract

CEs from C1–4 and dCE from Ultra and Xpert done on the serial dilutions were tested (heat extracted crude DNA from matching isolates was used as positive control). For each qPCR, 5 µl iTaq Universal SYBR Green Supermix (Bio-Rad), 0.3 µl (300 nM) of *M. tuberculosis* specific forward (V4 515F) primers, 0.3 µl (300 nM) of *M. tuberculosis* specific reverse (V4 806R) primers (Table S1) and 1.4 µl nuclease-free water was used^23^. 3 µl CE was added and amplification occurred using a Bio-Rad CFX-96. The threshold used to determine if a reaction was excluded from subsequent analyses was defined as a C_q_ value greater than the average of the triplicate negative controls for that run. Chambers with a C_q_ less than that average value were considered positive for *M. tuberculosis* complex (MTBC) DNA and used for MTBDR*plus*, MTBDR*sl* and FT.

### MTBDR*plus* and MTBDR*sl* line probe assays on cartridge extract

#### Diamond chamber extract

MTBDR*plus* and MTBDR*sl* (both version 2.0) were performed on dCEs from Ultra and Xpert done on the *in vitro* dilution series. For Ultra done on sputa from patients, only MTBDR*sl* was done. 5 µl dCE was used for MTBDR*plus* and MTBDR*sl* each. MTBDR*plus* and MTBDR*sl* results were reported as described^24^: either actionable [TUB-band positive and determinate (gene-specific locus bands present)] or non-actionable [TUB-band negative or TUB-band positive but indeterminate (gene-specific locus band absent)]. Susceptibility calls were made for all actionable results. Banding patterns were read by two experienced independent readers blinded to each other’s calls, the Ultra and Xpert results, and, for the dilution series experiement, the strain antibiograms (if there was a discrepancy between readers, a third experienced reader reviewed results and did the final classification).

#### Other chambers

MTBDR*plus* and MTBDR*sl* were done on C2 and C4 CEs from both Ultra and Xpert done on the dilution series. C1, C3, and C5 were not tested with LPAs as their CEs were 16S rRNA qPCR-negative or there was no volume remaining to test after the Ultra or Xpert test had completed (C5).

### FluoroType MTBDR on cartridge extract

#### Diamond chamber

dCEs from Ultra and Xpert cartridges done on the *in vitro* dilution series were tested by FT using the manufacturer’s instructions^25^. A total of 26 tubes for each test (Ultra, Xpert) were tested [four dilutions from 10^1^–10^4^ CFU/ml in triplicate for both strains plus a negative control for each strain, (4 × 3 × 2 + 2)]. As Xpert dCE had a volume of ~15 µl, after MTBDR*plus* (5 µl), MTBDR*sl* (5 µl), and the 16S rRNA qPCR (3 µl) were all done on the same Xpert dCE, the remaining volumes (5–14 µl) were made up to 20 µl with dH_2_O for FT (the recommended input volume)^25^. All Ultra dCEs (~35 µl originally) had 20 µl remaining and the full 20 µl was used for FT. FT results were classified in a manner similar to that for the line probe assays: actionable (MTBC detected; rifampicin and isoniazid susceptible or resistant) or non-actionable (no MTBC detected, MTBC indeterminate or MTBC detected but rifampicin or isoniazid indeterminate).

#### Other chambers

FT was done on C2 and C4 (as for LPAs) from both Ultra and Xpert cartridges used for the dilution series.

### Evaluation of *rpoB* amplicon cross-contamination risk

#### Amplicon escape during batched cartridge extractions

**Figure 3:**
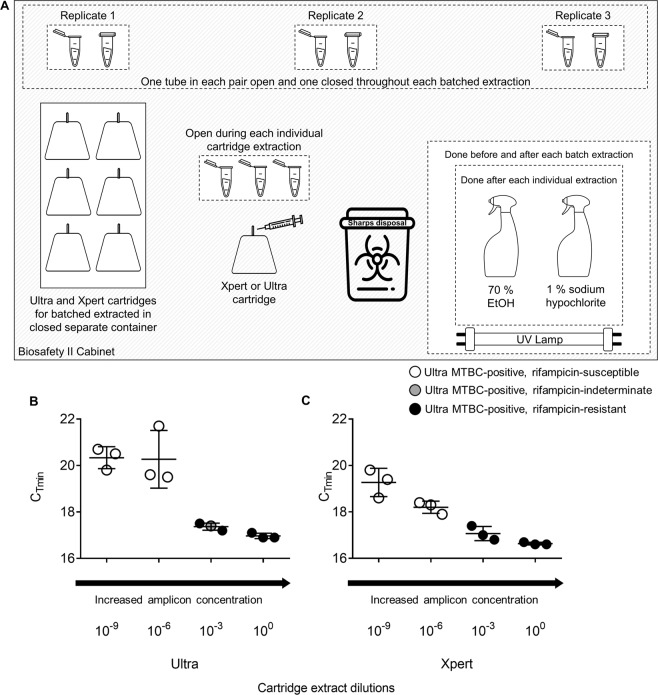
Evaluation of *rpoB* cross contamination risk experimental set-up and results. (**A**) Configuration of the environmental exposure experiment within a Biosafety level 2 cabinet. Three microcentrifuge tubes were open throughout each batched extraction procedure and three remained closed [median (IQR) extractions per batch 17.5 (10.5–27.5)]. No exposed tubes were FT *rpoB*-positive. In parallel to evaluate if, in an absolute worst case scenario, *rpoB* cross-contamination was probable, dCE from a (**B**) Ultra or (**C**) Xpert done on a drug-resistant strain was added to a drug-susceptible strain and the resultant mixture tested by Ultra. When samples of DS-TB contained CE at higher concentrations (undiluted, 10^−3^), false-resistant (solid black circles) or indeterminate rifampicin resistance (grey circles) are seen. All samples containing CE dilutions beyond 10^−6^ showed true rifampicin susceptibility (white circles). Error bars represent C_Tmin_ values for each dilution. Some images were obtained from the Noun Project: microcentrifuge tube (without changes), Anthony Ledoux, https://thenounproject.com/term/eppendorf/1699532/; spray bottle (without changes), John Winowiecki, https://thenounproject.com/search/?q=spray%20bottle&i=2236898; sharps container, Juicy Fish (with changes), https://thenounproject.com/term/hospital-waste-bin/2450390/; needle (without changes), Creative Mania; https://thenounproject.com/search/?q=injection&creator=2251916&i=2409865.

During all Ultra and Xpert diamond chamber extractions, 1.5 ml microcentrifuge tubes containing 100 µl sterile dH_2_O were positioned in the same BSL2 cabinet (Fig. 3A). Three tubes remained open throughout all extractions for each batch extraction and three remained closed (negative controls). Tubes were stored at −20 °C for later FT testing. A total of 20 batches of cartridges were extracted [n = 120 tubes in total from the 20 batches, n = 60 open tubes and n = 60 closed tubes including triplicates], with a median (IQR) number of cartridges per batch of 17.5 (10.5–27.5). There were also three tubes open for each individual cartridge extraction but these were not tested further based on results of the open tubes during batched extraction, which revealed no cross-contamination. Furthermore, extractions procedures were done by a total of five different users to reflect user variability.

#### Spiking of amplicons

The same XDR-TB strain with known Xpert and Ultra *rpoB* resistance profiles was used in the dilution series (Fig. 1C). Ultra and Xpert were each done on 1 ml of a 10^4^ CFU/ml concentration (in triplicate). dCEs were extracted and used for a dilution series (10^0^, 10^−3^, 10^−6^, and 10^−9^; each 1 ml final volume). For all dilutions, 5 µl was added to 700 µl of the DS-TB strain (10^4^ CFU/ml) and tested with Ultra [700 µl was used as, when combined with the recommended two-fold sample reagent volume, the 2 ml input volume is reached with minimal sample unused (~100 µl)].

## Results

### *Mycobacterium tuberculosis* complex genomic DNA detection in different chambers from cartridges done on dilution series

Though qPCR-positive results were obtained from C2, C4 and the dCE (Fig. S1), these results were highly variable even at high concentrations of bacilli (at least 10^4^ CFU/ml), suggesting interference. As C2, C4 and dCE gave positive qPCR results on cartridges done on some dilutions, and C1 and C3 gave none, we only explored the utility of the former for downstream testing using MTBDR*plus*, MTBDR*sl*, and FT.

### MTBDR*plus* and MTBDR*sl* on extract from cartridges done on dilution series

#### TB detection

**Figure 4:**
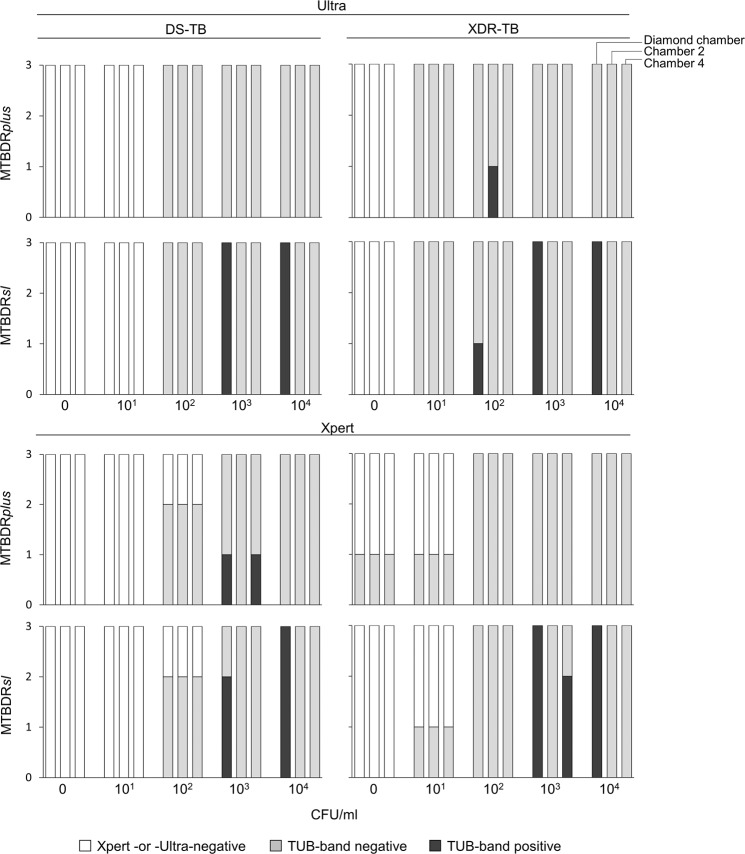
MTBDR*plus* and MTBDR*sl* on cartridge extract results for TB detection. dCE (left-most column), C2 (middle column) and C4 (right-most column) from *M. tuberculosis*-positive cartridges on dilution series (DS-TB and XDR-TB strains) are shown. MTBDR*plus* had mostly non-actionable results (not positive or negative). MTBDR*sl* had actionable results on all Ultra- and Xpert-positive dCE at >10^3^. Though some actionable line probe assay results for non-diamond chambers were observed, these were inconsistent and had low reproducibility.

More Ultras were MTBC-positive at lower CFU titres than Xpert [e.g., 4/6 (67%) of the 10^1^ CFU/ml aliquots vs. 1/6 (17%) for Xpert at the same concentration for both strains] (Fig. 4). MTBDR*plus* had high rates of non-actionable results across all dilutions irrespective of the cartridge chamber extract originated from (diamond, C2, C4) or initial test (Ultra, Xpert) (Fig. 4). MTBDR*sl* had actionable results for all Ultra dCEs ≥10^3^ CFU/ml and, for Xpert, all but one dCE ≥10^3^ CFU/ml (one Xpert replicate at 10^3^ CFU/ml was MTBDR*sl*-non-actionable). MTBDR*sl* on C2 and C4 had non-actionable results across all dilutions (Ultra and Xpert).

#### Resistance detection

**Figure 5:**
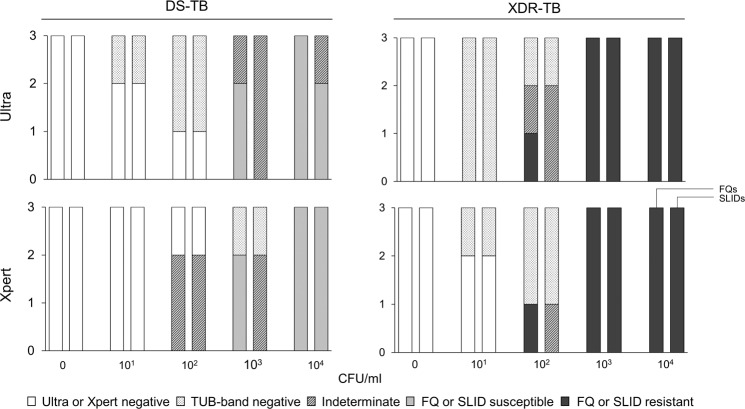
MTBDR*sl* drug susceptibility results on dCEs from Ultra and Xpert on dilution series. All results ≥10^3^ CFU/ml for the XDR-TB strain had resistance results concordant with the isolate. Some SLIDs indeterminate results were seen for the DS-TB >10^3^ at the same concentrations but MTBDR*sl* results were otherwise concordant with those on the isolate.

MTBDR*sl* correctly identified FQ and SLID resistance on Ultra dCE done on all XDR strain aliquots ≥10^3^ CFU/ml (Fig. 5). On the DS-TB strain, MTBDR*sl* identified FQ susceptibility in all three 10^4^ CFU/ml replicates and in 2/3 (67%) replicates for SLIDs (one indeterminate). At 10^3^ CFU/ml for the DS-TB strain, 2/3 (67%) were correctly identified as FQ susceptible (one indeterminate) and all were SLID-indeterminate. The C_Tmin_ threshold at which all MTBDR*sl* results was feasible on Ultra CE was <25, which was used for further experiments. Similar results were obtained for MTBDR*sl* on Xpert dCE.

### MTBDR*sl* on extract from cartridges done on clinical specimens

#### TB detection

As MTBDR*plus* was not feasible in the *in vitro* assessment, it was not done on CE from Ultras done on clinical sputa. MTBDR*sl* on dCE from Ultra done on clinical sputa had 37/40 (93%) actionable results (the rest were non-actionable). Non-actionable results corresponded to “trace” or “very low” semi-quantitative categories.

#### Resistance detection

Of the actionable results, 35/37 (95%) fell within the defined threshold (C_Tmin_ <25) and of these all FQ results were concordant with MTBDR*sl* on sputum and all but one SLID result were concordant (false-susceptible). Though this percentage is slightly higher than the number of patients with C_Tmin_ <25 in our setting, which was determined to be 86% (based on an evaluation of Ultra done in sympotmatic patients in primary care^26^), which further show that this approach would benefit the majority of patients in our setting. Of the 2/37 (5%) results that were actionable but fell above the defined threshold, one was concordant with MTBDR*sl* on sputa and one was indeterminate for FQs and discordant for SLIDs (false-resistant).

#### Receiver operator curve for determining actionable results

**Figure 6:**
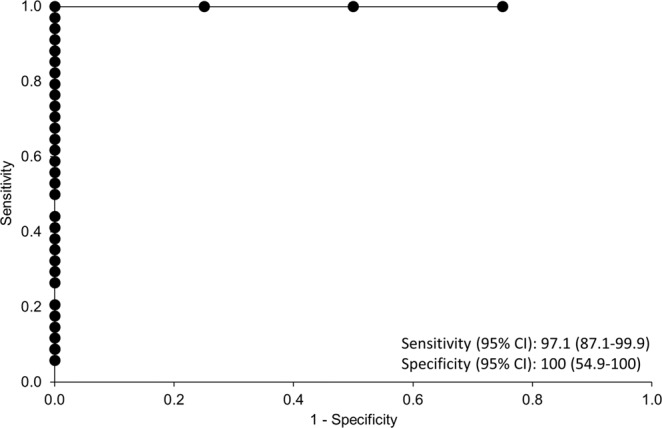
Receiver operation area under the curve of actionable vs. non-actionable results of MTBDR*sl* on Ultra diamond cartridge extract done on DR-TB clinical sputa to determine a C_Tmin_ threshold at which this approach is not feasible. MTBDR*sl* yields actionable results on cartridge extract from Ultra at a C_Tmin_ threshold of <25.4 with a sensitivity of 97% (87.1–99.9; 95% CI) and specificity of 100% (54.9–100; 95% CI) respectively.

An Ultra *rpoB* C_Tmin_ threshold of <25.4 was defined for dCEs done on clinical sputa with sensitivities of 97% (95% CI 87–100) and specificities of 100% (55–100) (Fig. 6).

### FluoroType MTBDR on extract from cartridges done on dilution series

#### Diamond chamber

FT had similar results to MTBDR*plus* on CEs. For example, 3/24 (12%) Ultra dCEs were MTBC-positive (the others negative) for both strains (Fig. S2). In the three Ultra dCEs with a TB-positive FT result, all had indeterminate susceptibility results for at least one drug. A total of 18/24 (75%) Xpert dCEs were FT MTBC-positive, however, of these 13/24 (54) were indeterminate for at least one drug.

#### Chamber 2

FT on Ultra C2 had MTBC positivity rates of 10/12 (83%) and 11/12 (92%) for DS-TB and XDR-TB, respectively. On Xpert C2, FT TB positivity rates were 5/12 (42%) and 7/12 (58%) for DS-TB and XDR-TB, respectively. In MTBC-positive extracts (Ultra and Xpert), most resistance calls were indeterminate or discordant with the paired isolate.

#### Chamber 4

FT done on C4 from Ultra had 8/12 (67%) and 9/12 (75%) TB positivity rates for DS-TB and XDR-TB strains respectively, and 3/12 (25%) and 1/12 (8%) on for C4 from Xpert respectively. As for C2, resistance calls were mainly indeterminate or discordant with paired isolate.

### *rpoB* amplicon cross-contamination risk evaluation

#### Exposure of open tubes during batched extractions

All sixty tubes exposed were FT MTBC-negative and had no *rpoB* amplification.

#### Amplicon spiking for absolute worst-case cross-contamination scenario

Of the Ultra dCEs done on XDR-TB and spiked into DS-TB for re-testing with Ultra, evidence of cross-contamination was seen when dCEs were diluted less than 10^−6^ before addition to the DS-TB strain [3/3 (100%) of 10^0^ dilutions and 2/3 (67%) of the 10^−3^ dilutions showed false-resistance (1/3 of the 10^−3^ was resistance indeterminate)] (Fig. 3B). Similar results were obtained for Xpert dCE (Fig. 3C).

## Discussion

We have validated MTBDR*sl* on CEs from used Ultra cartridges for genotypic second-line DST. We show: (1) MTBDR*sl* on Ultra dCE when C_Tmin_ <25 enabled DST concordant with sputum results, (2) risk of *rpoB* extract cross-contamination is unlikely if standard aseptic protocols are followed, (3) neither 16S rRNA qPCR, MTBDR*plus*, MTBDR*sl* nor FT are feasible on other cartridge chambers, nor was MTBDR*plus* or FT on Ultra and Xpert dCEs. These data support the use of Ultra extract for second-line genotypic DST.

We defined a threshold at which MTBDR*sl* is likely to work on Ultra dCE from the vast majority of Ultra-positive patients, thereby avoiding time and resources wasted on dCE unlikely to give a valid result. We are mindful that there were some indeterminate SLID results (in line with previous reports of higher MTBDR*sl* indeterminate result rates for SLIDs vs. FQs)^27–29^. However, all dCE SLID-indeterminate results from the dilution series were from the DS-TB strain and there were no indeterminate SLID results on XDR-TB dCEs. On clinical sputum (and falling within our threshold), one MTBDR*sl* SLID susceptibility result was discordant with sputum (one false-negative). We thus suggest that MTBDR*sl* Ultra dCE results are interpreted in the same manner as recommended by the WHO for MTBDR*sl* on clinical specimens^30^. If, for example, MTBDR*sl* on dCE is non-actionable or susceptible, MTBDR*sl* on sputum or isolates should be done. If there is still no evidence of resistance in a high burden setting, phenotypic DST should still be done given the suboptimal rule-out accuracy of MTBDR*sl*^19,30^.

The possibility of contamination from *rpoB* amplicons during extractions has not been investigated. We implemented systematic testing for possible environmental contamination. No tubes exposed for each extraction batch were *rpoB*-positive when tested with FT. FT was used for testing for *rpoB* amplicons as it is more sensitive than MTBDR*plus*^14,15^.

We further tested a worst-case contamination scenario with dCEs from both Ultra and Xpert cartridges done on a XDR-TB strain, diluting these dCEs, and adding them to a DS-TB strain which was subsequently tested by Ultra. The undiluted and most concentrated dCE dilutions (10^0^_,_ 10^−3^) showed false rifampicin-resistance indicating that, although the GeneXpert platform does have proven ability to remove large numbers of amplicons^31^, it was not able to remove all amplicons during the pre-amplification wash steps, however, amplicons diluted beyond 10^−3^ were successfully removed to the point of not being detected^22,32,33^. These results, together with those from the environmental samplings during extractions, shows that when standard aseptic techniques are used, amplicon cross-contamination is highly unlikely except in the artificial worst case scenarios. Finally, it should be noted that, in line with good practice in any molecular biology laboratory providing results for patient management, dCEs should not be collected in the same room where *rpoB-*based tests are done, and that the risk of cross-contamination from the dCE approach is only pertinent to tests for rifampicin resistance.

We suggest that diagnosticians considering implementing this approach use the cartridge  itself as a transport vessel (upright and in sealed containers) to a central laboratory where dCE can be extracted appropriately (the diamond is a sealed chamber and should remain safe during transport). Most peripheral laboratories will be unable to do the dCE procedure safely and downstream molecular DST like MTBDR*sl*. This cartridge transport can interface with existing specimen referral networks. If dCE is planned purely for molecular epidemiology, we suggest that dCE be extracted and stored at −80 °C or alternatively the whole cartridge be stored at −20 °C until extractions can be done in a batched, centralised fashion. The long term stability of these approaches will require examination.

We further hypothesised that liquid from other cartridge chambers may avoid interference by *rpoB* amplicons. However, upon testing, this approach gave variable non-replicable results. This was true for qPCR, MTBDR*plus*, MTBDR*sl* and FT assays. This may also be due to very low concentrations of template in these chambers, for example C3 – which is the “wash chamber”, and/or remnant PCR inhibitors (e.g., salts from the sample reagent). In light of this, we believe that the presence of these amplicons may prevent newer approaches, such as next generation sequencing methods, from performing well on dCE without to clean up steps. This warrants further investigation. CE from the diamond chamber hence remains the best option for downstream genotypic DST.

The results of this study should be interpreted within its limitations, namely aseptic techniques done in an assay- or procedure-specific biosafety cabinet are needed to minimise amplicon cross-contamination. However, this infrastructure should already be implemented per WHO guidelines^34^ where LPAs are done routinely for patient care. Furthermore, per good laboratory practice, CEs should not be collected in the same room where *rpoB-* or *IS*6110*/*1081-based assays are done, nor should either procedure be done by the same personnel on a daily basis. Lastly, further investigation into cross-contamination risk should be done in a routine diagnostic setting. This should include multiple operators.

We also acknowledge that this method may increase risk of needle stick injury. Standard biosafety protocols should be strictly adhered to. We were recently funded to develop a device that can eject material from cartridges in a safe manner. Another limitation is MTBDR*plus* was not feasible on Ultra CEs and we suspect this is due to interference from both *rpoB* and IS*6110*/*1081* amplicons. Thus, combined with the large volumes (and hence diluted targeted DNA) recovered from non-diamond chambers in Ultra and Xpert, MTBDR*plus* (and also likely FT) on extract from any Ultra cartridge chamber is in all likelihood not useful for isoniazid or confirmatory rifampicin DST. Finally, although the diamond chamber is a closed system and appears protected against desiccation, we acknowledge that some desiccation may occur over prolonged periods that this requires future systematic evaluation. However, we recommend that extract method is done on an as fresh a cartridge as possible (either at a peripheral or central laboratory), in order to reduce the delays of DR-TB diagnosis. Formal evaluation of CE stability pre-extraction may be useful.

We conclude that dCEs from Ultra at the C_Tmin_ threshold (<25), can be used for genotypic second-line DST (MTBDR*sl*). Ultra and MTBDR*sl* on dCE therefore allows for the rapid rule-in detection of XDR-TB on a single specimen.

## Supplementary information

Supplementary information

## Data Availability

The datasets generated during and/or analysed during the current study are available from the corresponding author on request.
