# Supplementary material for: Extract from used Xpert MTB/RIF Ultra cartridges is useful for accurate second-line drug-resistant tuberculosis diagnosis with minimal rpoB-amplicon cross-contamination risk
Source: Sci Rep. 2020 Feb 14;10:2633. doi: 10.1038/s41598-020-59164-3 (PMC7021780; doi:10.1038/s41598-020-59164-3)
Supplement: Supplementary file 1 — Supplementary information [file 41598_2020_59164_MOESM1_ESM.pdf]

Extract from used Xpert MTB/RIF Ultra cartridges is useful for accurate second-line drug-resistant tuberculosis diagnosis with minimal *rpoB*-amplicon cross-contamination risk

Rouxjeane Venter[1+], Stephanie Minnies[1+], Brigitta Derendinger[1], Happy Tshivhula[1], Margaretha de Vos[1], Tania Dolby[2], Ashley Ruiters[2], Robin M. Warren[1], Grant Theron[1*]

[1]DST/NRF Centre of Excellence for Biomedical Tuberculosis Research, SA MRC Centre for Molecular and Cellular Biology, Division of Molecular Biology and Human Genetics, Faculty of Medicine and Health Sciences, Stellenbosch University

[2] National Health Laboratory Services, Cape Town, South Africa.

*Address correspondence to A/Prof Grant Theron: gtheron@sun.ac.za.

[+]Contributed equally.

Supplementary Tables:…………………………………………………………………...3

Table S1: V4 region *Mtb* specific 16S rRNA primers and thermocycling conditions

Supplementary Figures:……………………………………………………………..4

Figure S1: Quantitative PCR amplification results on cartridge extract (CE) from both Xpert and Ultra cartridges done on both DS-TB and XDR-TB on a dilution series (10^0 – 10^4 CFU/ml). The 16s rRNA gene was amplified in some diamond CEs, C2, and C4 replicates; however, results were inconsistent……………………………………….4

Figure S2: FluoroType MTBDR results on Ultra and Xpert diamond cartridge extract (CE) done on either a DS-TB or XDR-TB dilution series. Left bars indicate rifampicin and right bars indicate isoniazid. Most results were non-actionable. More Ultra results were "MTBC not detected" compared to Xpert CE results……………………………5

Supplementary methods:……………………………………………………...……..6

Cartridge structure and design ……………………………………………………...6

References:…………………………………………………………………………7

Table S1: V4 region *Mtb* specific 16S rRNA primers and thermocycling conditions

Mtb Forward primer	5’-GTGCCAGCAGCCGCGGTAA-3’
Mtb Reverse primer	5’-GGACTACCAGGGTATCTAAT-3’
Thermocycling conditions<br>(Bio-Rad CFX-96 Real-Time PCR machine)	95°C for 5 minutes<br>35 cycles of 95°C for 5 seconds<br>60°C for 30 seconds.

Figure S1

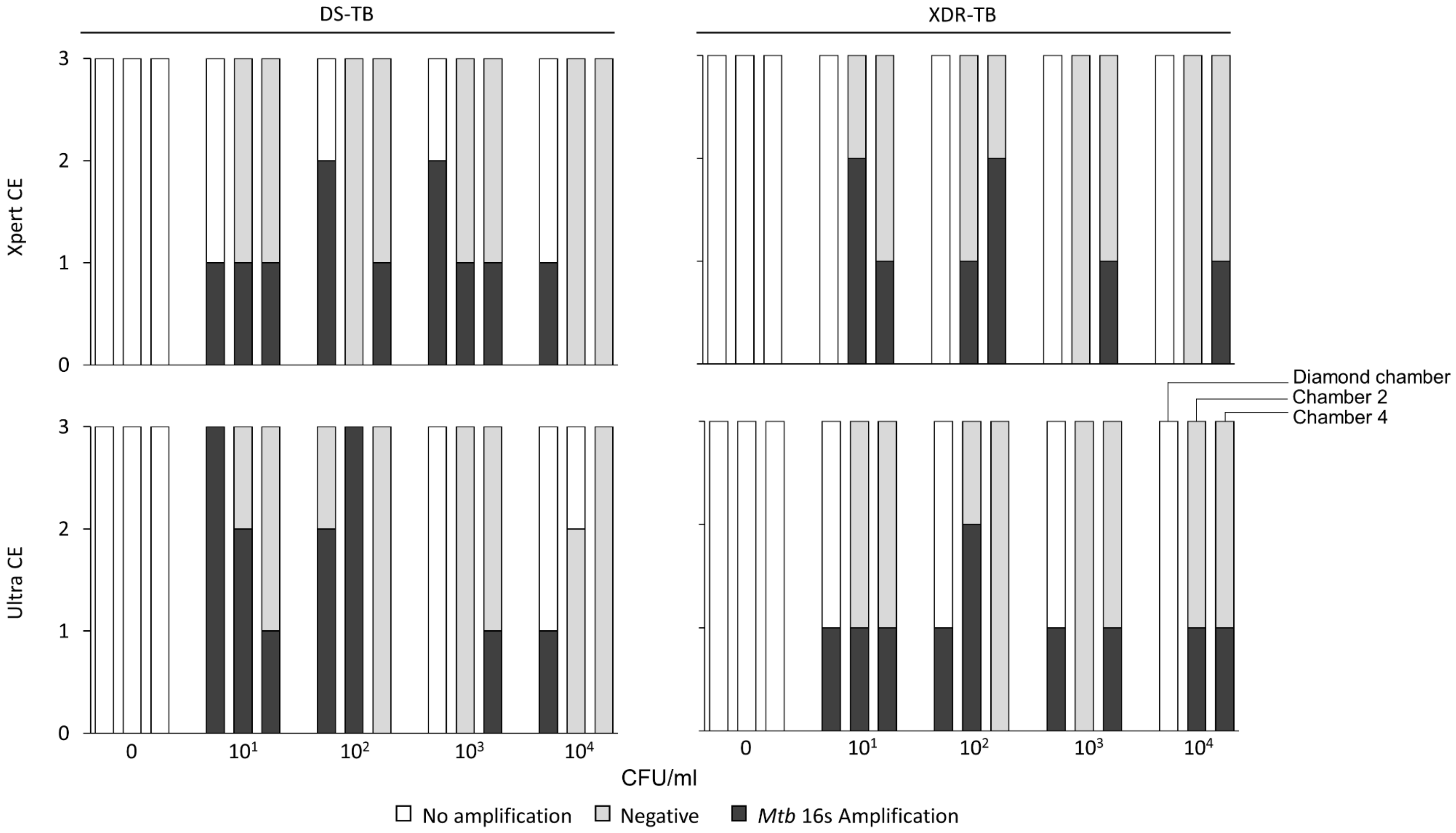
DS-TB
XDR-TB
Xpert CE
Ultra CE
0
1
2
3
0
10^1
10^2
10^3
10^4
CFU/ml
Diamond chamber
Chamber 2
Chamber 4
No amplification
Negative
Mtb 16s Amplification

Figure S2:

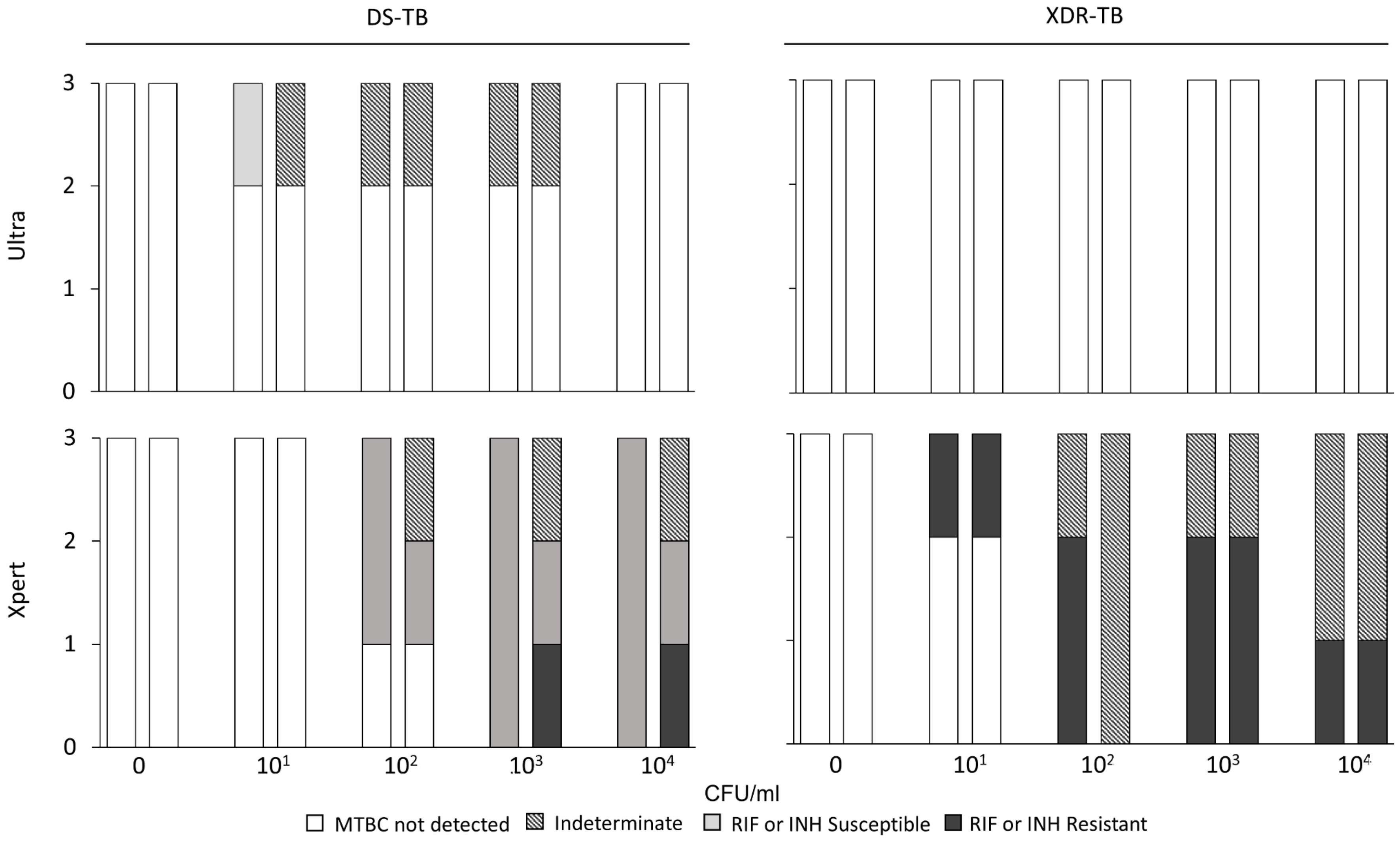
DS-TB
XDR-TB
Ultra
Xpert
0
1
2
3
0
10^1
10^2
10^3
10^4
CFU/ml
MTBC not detected
Indeterminate
RIF or INH Susceptible
RIF or INH Resistant

Cartridge structure and design

First, sample reagent is mixed with the specimen and 2 ml pipetted into the cartridge [Chamber 1 (C1); ~500 µl typically remaining after test completion]. The cartridge lid is then closed before placement in the GeneXpert machine and the test started. A plunger is automatically inserted into the cartridge centre. This engages a barrel to sequentially draw buffers and reagents into the chambers[1,2]. The plunger first draws the sample mixture into the cartridge base where bacilli (not necessarily alive or intact) are trapped on a filter prior to DNA extraction (Figure 2A)[3]. Wash fluid in Chamber 2 (C2, ~3 ml remaining), is then drawn over the filter and waste is collected in Chamber 3 (C3, ~5 ml remaining). While this wash can mostly (but not completely) remove small DNA fragments, it does not remove large DNA, especially if debris-associated[3-5]. Liquid reagent is then drawn from Chamber 4 (C4, ~500 µl remaining) and a sonic horn applied to lyse bacteria and release DNA[6]. DNA is then drawn into Chamber 5 (C5, no volume remaining) and mixed with reaction beads. This mixture is channelled into the reaction chamber (diamond protrusion at the back of cartridge where dCE is drawn from) where thermocycling and PCR amplification takes place[6].

References:

1 Raja, S. *et al.* Technology for automated, rapid, and quantitative PCR or reverse transcription-PCR clinical testing. *Clinical chemistry* **51**, 882-890 (2005).

2 Niemz, A., Ferguson, T. M. & Boyle, D. S. Point-of-care nucleic acid testing for infectious diseases. *Trends in biotechnology* **29**, 240-250 (2011).

3 Theron, G. *et al.* Xpert MTB/RIF results in patients with previous tuberculosis: can we distinguish true from false positive results? *Clinical Infectious Diseases* **62**, 995-1001 (2016).

4 Theron, G. *et al.* False positive Xpert MTB/RIF results in re-tested patients with previous tuberculosis: frequency, profile, and prospective clinical outcomes. *Journal of clinical microbiology*, JCM. 01696-01617 (2018).

5 Blakemore, R. *et al.* Evaluation of the analytical performance of the Xpert MTB/RIF assay. *Journal of clinical microbiology* **48**, 2495-2501 (2010).

6 Cepheid. *Journey Inside the Cepheid GeneXpert® Cartridge - 3D Animation*, 2019).
